# Design and Implementation of a Composite Hydrophone of Sound Pressure and Sound Pressure Gradient

**DOI:** 10.3390/mi12080939

**Published:** 2021-08-10

**Authors:** Guojun Zhang, Lansheng Zhang, Songxiang Ji, Xi Yang, Renxin Wang, Wendong Zhang, Shie Yang

**Affiliations:** 1 Key Laboratory of National Defense Science and Technology for Underwater Acoustic Technology, Harbin Engineering University, Harbin 150001, China; yangshie@hrbeu.edu.cn; 2 State Key Laboratory of Dynamic Testing Technology, North University of China, Taiyuan 030051, China; zhanguojun1977@nuc.edu.cn (S.J.); m18434363051@outlook.com (X.Y.); wangrenxin@nuc.edu.cn (R.W.); wdzhang@sxedu.gov.cn (W.Z.); 3 ZJU-UIUC Institute, International Campus, Zhejiang University, Haining 314400, China

**Keywords:** MEMS, cilium, composite hydrophone, left–right ambiguity, sensitivity, directivity

## Abstract

The bionic cilium MEMS vector hydrophone has the characteristics of low power consumption, small volume, and good low-frequency response. Nevertheless, there exists the problem of left–right ambiguity in the azimuth estimation of a single hydrophone. In order to solve the engineering application problem, a sound-pressure sound-pressure-gradient hydrophone is designed in this paper. The new composite hydrophone consists of two channels. The bionic cilium microstructure is optimized and used as the vector channel, to collect the sound pressure gradient information, and a scalar channel, based on a piezoelectric ceramic tube, is added, to receive the sound pressure information. The theoretical analysis, simulation analysis, and test analysis of the composite hydrophone are carried out, respectively. The test results show that the sensitivities of the hydrophone can reach up to −188 dB (vector channel) and −204 dB (scalar channel). The problem of left–right ambiguity is solved by combining the sound pressure and sound pressure gradient in different ways. This is of great significance in the engineering application of single cilium MEMS hydrophone orientation.

## 1. Introduction

With the development of underwater acoustic technology, many hydrophones, based on different principles, have been designed [1,2,3,4,5], such as the fiber laser hydrophone, piezocomposite hydrophone, and electromagnetic hydrophone, etc. Chen, H et al. proposed a compact fiber-optic hydrophone and used it for acoustic wave measurements [6]. Xu, JH et al. presented an experimental investigation of a water pipeline leak detection system, based on a low-cost, tiny-sized hydrophone sensor that was fabricated using the MEMS technologies [7]. A molecular-electronic hydrophone, with a frequency range of 0.02 Hz−200 Hz and sensitivity of 0.75 mV/Pa, was described, to measure ambient noise with the use of correlation analysis [8]. A vector hydrophone was developed by Pyo, S et al., for a towed array sonar system, using a shear type accelerometer. The vector hydrophone has the highest feasible receiving voltage sensitivity (RVS) over the desired frequency range, in comparison with other types of vector hydrophones, such as the multimode hydrophone [9].

The vector hydrophone has many advantages [10,11], which not only can measure the vector information, such as the pressure gradient, velocity, acceleration, and acoustic energy flow, but can also suppress co-directional environmental noise and improve the anti-jamming ability of the system. Jin, MQ et al. proposed a three-component homovibrational vector hydrophone, with a low-reflection fiber Bragg grating (FBG). The test results showed that the working band of the hydrophone was extended to 20 Hz, and the sensitivity of each vector channel was about −132.5 dB at 500 Hz, and its directivity index was more than 30 dB at 100 Hz [12]. Zhang, WT et al. presented a three-axis slim fiber laser vector hydrophone. The experimental results showed an acceleration sensitivity of 53.2 pm/g, 39.2 pm/g, and 38.1 pm/g at the *x*, *y*, *z* axis, respectively, a resonant frequency of about 310 Hz, and a directivity that was larger than 30 dB [13].

The piezoelectric ceramic transducer is widely used in the underwater acoustics field, which possesses many good properties [14,15], such as a simple structure, stable working characteristics, high receiving sensitivity, and convenient array arrangement. A needle hydrophone was designed by using PMN–PZT piezoelectric single crystal for application to several MHz photoacoustic imaging systems [16]. However, the hydrophone based on piezoelectric devices can only detect the scalar information of sound pressure. For underwater target location, an array is usually needed.

A bionic cilium MEMS vector hydrophone (CVH), with a sensitivity of −197 dB, a resonant frequency of 1 kHz, proposed by North University of China, has the characteristics of low power consumption, small size, and good low-frequency response [17]. However, for CVH, there exists the problem of left–right ambiguity in the azimuth estimation of a single hydrophone. Combining scalar information and vector information is undoubtedly an excellent choice to improve the accurate positioning ability. Fu JW et al. constructed a composite vector hydrophone, with discrete velocity hydrophones and several pressure hydrophones, according to a specific spatial distribution. The velocity hydrophones acquired particle velocity information of the underwater sound field, and the pressure hydrophones received underwater sound pressure signals [18]. Liu MR et al. designed a composite MEMS hydrophone, which consists of an acoustic pressure gradient bionic cilium MEMS vector hydrophone and a capacitive MEMS acoustic pressure hydrophone [19]. In this paper, a composite hydrophone was designed by adding a piezoelectric ceramic tube to a bionic cilium MEMS hydrophone. The vector information and scalar information that were combined in different ways, realized the underwater positioning of a single hydrophone.

## 2. Directional Analysis

**Figure 1:**
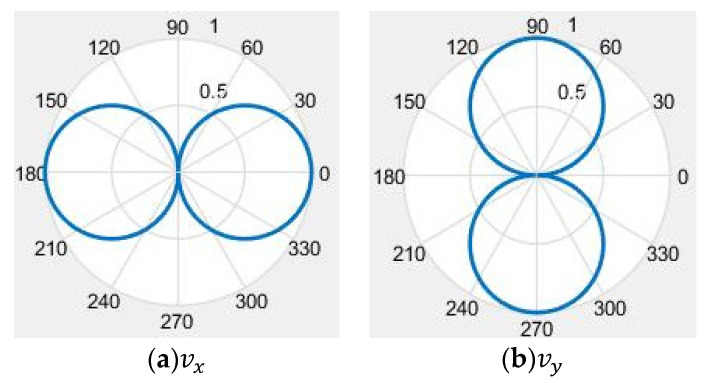
Directivity of cilium MEMS vector hydrophone. (**a**) The directivity in *x* direction; (**b**) the directivity in *y* direction.

In ideal fluid medium, the relationship between sound pressure gradient and vibration velocity, at any point, can be expressed as Formula (1) [19], as follows:

(1) $$\rho_{0}\frac{\partial \vec{v}}{\partial t}=-\nabla p$$

wherein, $\rho_{0}$ is medium density; $\vec{v}$ is particle velocity; ∇*p* is acoustic pressure gradient. For two-dimensional directivity in plane, the received information, by the cilium hydrophone, can be expressed as Formula (2), as follows:

(2) $$v_{x}\left(t\right)=x_{t}\cos\theta \;v_{y}\left(t\right)=x_{t}\sin\theta$$

wherein, $v_{x}$ and $v_{y}$ are the particle velocity information in the *x* and *y* directions, respectively; *t* is time; *θ* is the angle between the particle in the horizontal plane and the positive direction of the *x*-axis. The simulation diagrams of directivity are shown in Figure 1. Figure 1a,b show the directivity in the *x* and *y* direction, respectively. It shows that the cilium MEMS vector hydrophone has a dipole directivity, but there exists left and right ambiguity when estimating the target direction.

**Figure 2:**
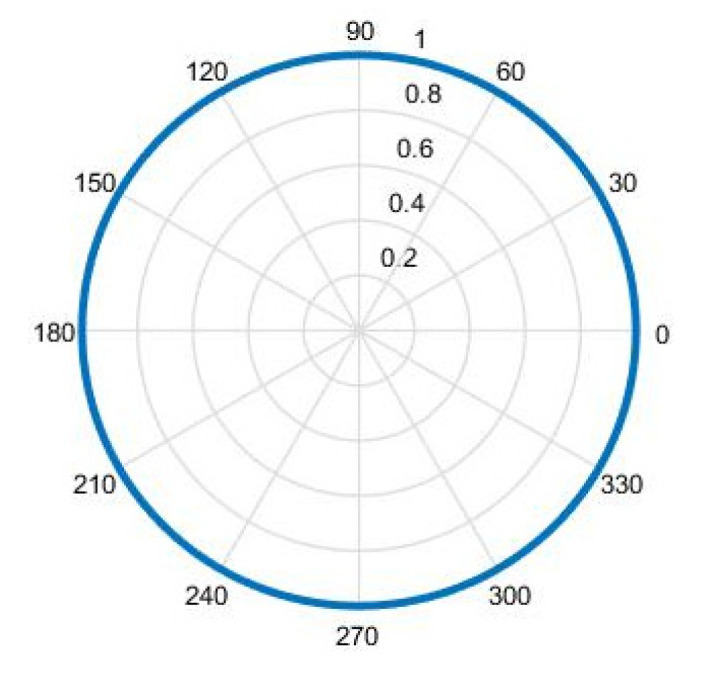
Directivity of the piezoelectric tube.

The piezoelectric tube can receive sound pressure information, but it has no directivity in the horizontal direction. The sound pressure information that was received by the piezoelectric tube is expressed as Formula (3), and the directivity simulation diagram is shown in Figure 2.

(3) p(t)=x(t)

wherein, *p*(*t*) is the sound pressure information.

**Figure 3:**
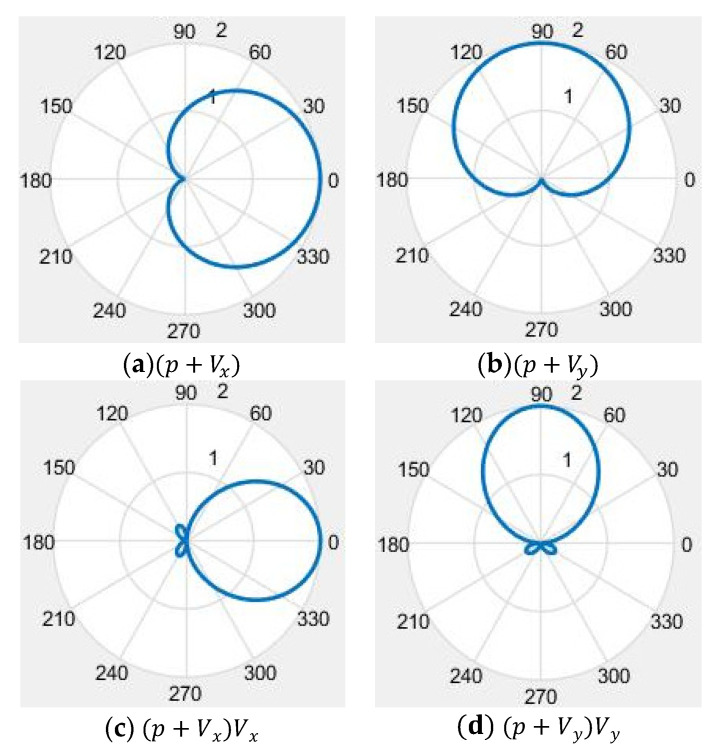
Directivity of the composite hydrophone. (**a**,**b**) Sound pressure and vibration velocity are combined by the mode of $p+V_{i}$(i=x,y); (**c**,**d**) sound pressure and vibration velocity are combined by the mode of $\left(p+V_{i}\right)V_{i}$ (i=x,y).

**Figure 4:**
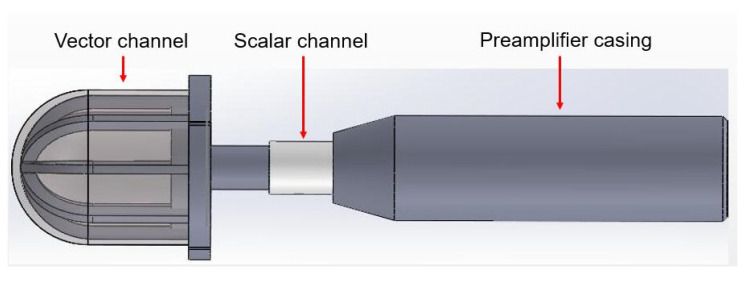
Schematic diagram of the composite hydrophone.

From Figure 1 and Figure 2, we can see that neither the single cilium MEMS vector hydrophone nor the piezoelectric tube transducer can achieve underwater positioning separately. In this paper, a composite hydrophone that was designed, used the cilium MEMS vector hydrophone as the vector channel and the piezoelectric circular tube as the scalar channel. The composite hydrophone can not only receive velocity information, but also synchronously receive sound pressure information in the sound field. As shown in Figure 3, when sound pressure and vibration velocity are combined in $\left(p+V_{i}\right)$ and $\left(p+V_{i}\right)V_{i}\;\left(i=x,y\right)$ modes, they have different unidirectional directivity. The schematic diagram of the composite hydrophone is shown in Figure 4. There are the following three parts: vector channel, scalar channel, and preamplifier casing, where the signal processing PCB lies. The vector channel is a cilium bionic MEMS microstructure, wrapped in a cap filled with silicone oil.

## 3. Design of the Composite Hydrophone

### 3.1. Design of Vector Channel

**Figure 5:**
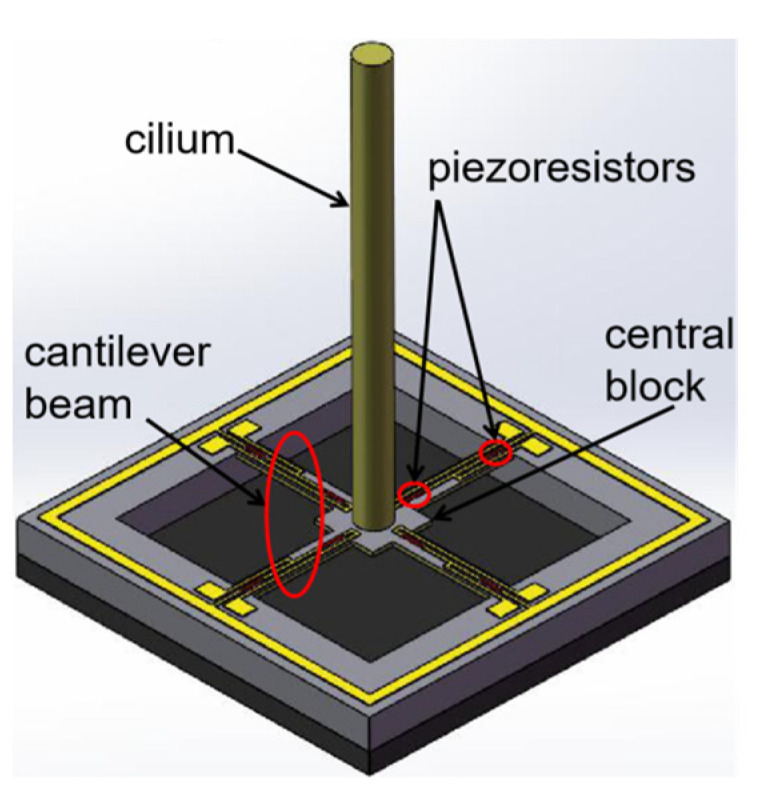
Schematic diagram of vector channel microstructure.

**Figure 6:**
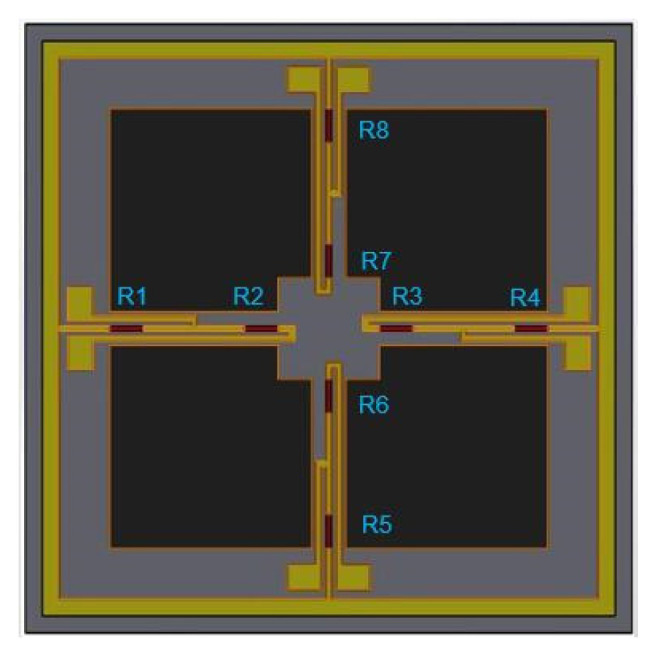
The distribution diagram of piezoresistors on beams.

The vector channel is a bionic cilium MEMS vector hydrophone. The sensor microstructure consists of the following three parts: cilium, four cantilever beams, and central block, as shown in Figure 5. When underwater, the acoustic signal acts on bionic cilium, and the cilium fluctuation is transmitted to cantilever beams, which produces piezoresistor deformation on the cantilever beams. Ultimately, the piezoresistor change is transformed into a voltage change and output through the Wheatstone bridge. The piezoresistors are arranged on the cantilever beams, which is shown in Figure 6. There are eight identical piezoresistors. *R_1_, R_2_, R_3_, R_4_* and *R_5_, R_6_, R_7_*, and *R_8_* form two Whiston bridges, respectively. The voltage output of the Whiston bridge can be expressed as Formula (4) [20], as follows:

(4) $$V_{out}=\frac{\Delta R}{R}V_{in}$$

wherein, $V_{out}$ is voltage output; $V_{in}$ is voltage input; ΔR is piezoresistor change; *R* is the initial value of piezoresistor.

### 3.2. Sensing Principle of Scalar Channel

**Figure 7:**
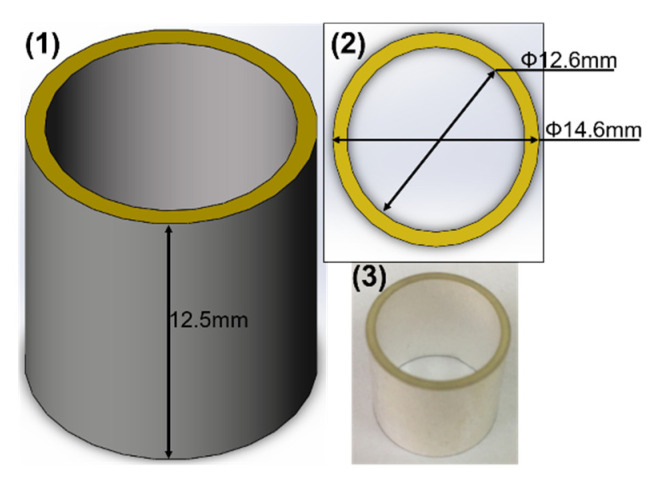
The piezoelectric ceramic tube. (**1**) A model in isometric view orientation; (**2**) in top-view orientation; (**3**) physical picture of piezoelectric tube.

The scalar channel is completed by the piezoelectric ceramic tube transducer. When the sound pressure acts on the polarized piezoelectric ceramic tube, due to the certain consistency in the direction of domain polarization intensity, the deformation of the piezoelectric ceramic tube will make the polarization intensity converge along the same direction. At this time, there will be charge accumulation on the electrode surface, which produces the piezoelectric effect. The inner wall of the piezoelectric ceramic tube is used as the ground and the outer wall is used as the terminal, to realize the sound pressure detection of the scalar channel. Figure 7 shows a physical picture and the dimensions of the piezoelectric ceramic tube.

## 4. Theoretical Analysis

### 4.1. Sensitivity and Natural Frequency Analysis of Vector Channel

The free field sensitivity indicates the induced electrical signal when it receives the unit sound pressure signal. For the vector channel, the voltage output is expressed in Formula (4). When the coordinate axis is consistent with the crystal axis, the relationship between the varistor relative change and stress on the beams is shown in Formula (5) [20], as follows:

(5) $$\frac{\Delta R}{R}=\pi_{l}\sigma_{l}+\pi_{t}\sigma_{t}+\pi_{s}\sigma_{s}$$

wherein, $\pi_{l}$, $\pi_{t}$ and $\pi_{s}$ are, respectively, the longitudinal, transverse and vertical piezoresistance coefficients; $\sigma_{l}$, $\sigma_{t}$ and $\sigma_{s}$ are, respectively, the longitudinal stress, transverse stress and vertical stress. As $\;\sigma_{s}$ is much smaller than $\sigma_{l}$ and $\sigma_{t}$, it can be ignored. Furthermore, in the design process, the longitudinal stress is increased as much as possible, and the transverse stress is restrained. The $\sigma_{t}$ is much smaller than $\sigma_{l}$, hence it can be neglected too. Formula (5) is approximated as follows:

(6) $$\frac{\Delta R}{R}=\pi_{l}\sigma_{l}\;$$

The vector channel designed in this paper is improved on CVH, and the sizes comparison is shown in Table 1. Combining theoretical mechanics analysis, elasticity analysis, and static analysis, it can be concluded that the stress at any point on the cantilever beams is as follows [21]:

(7) $$\sigma_{(x)\;}=\pm \;4pDH\;\left[\;\frac{L^{2}+3aL-3x\left(a+L\right)}{\frac{2}{3}bt^{2}\left(L^{2}+3aL+3a^{2}\right)}\left(\frac{1}{2}H\right)\pm \frac{1\;}{bt\;}\right]\;$$

wherein, *p* is external pressure; *L* is the length of beam; *a* is the half length of center block’s side; *b* is the width of beam; *t* is the thickness of beam; *D* and *H* are the diameter and height of cilium, respectively. Formula (7) shows that the height and diameter of cilium promote the sensitivity.

As another important parameter of the hydrophone, the natural frequency determines the application range of the hydrophone. According to previous studies, the natural frequency of the bionic cilium vector hydrophone can be expressed as follows (8) [22]:

(8) $$f=\frac{1}{2\pi }\sqrt{\frac{K}{m}}=\frac{1}{2\pi }\sqrt{2\frac{Ebt^{3}}{mLh^{2}}\left(\frac{a^{2}}{L^{2}}+\frac{a}{L}+\frac{1}{3}\right)}\;$$

wherein, *K* and *E* are the rigidity and elasticity modulus of cantilever beams, respectively; *m* is the mass of cilium; other parameters are the same as Formula (4). From Formula (8), it can be concluded that the cilium diameter promotes the natural frequency, while the cilium length inhibits it. In order to increase the sensitivity as much as possible, while maintaining the frequency bandwidth, the cilium diameter must be kept within a suitable range (200 μm−600 μm).

### 4.2. Scalar Channel Size Determination

The sensitivity of the piezoelectric ceramic tube is related to the material, and inner and outer diameter of the tube [23]. In this paper, the material of the piezoelectric ceramic tube is PZT−4. Owing to the fact that the piezoelectric ceramic tube is mounted on the metal shell of the cilium MEMS vector hydrophone, the dimensions of the inner radius are limited. There is a proportional relationship between the sensitivity of the piezoelectric ceramic tube and the wall thickness [23]. According to the processing difficulty, the wall thickness is set to 1 mm. The size of the piezoelectric ceramic tube is determined, which has a height of 12.5 mm, and an inner and outer radius of 6.3 mm and 7.3 mm, respectively.

## 5. Simulation Analysis

### 5.1. Stress Analysis on Beams of Vector Channel and Sensitivity Analysis of Piezoelectric Tube

**Figure 8:**
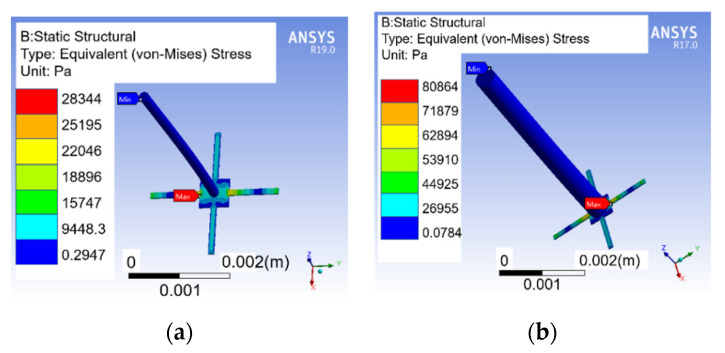
Stress distribution on cantilever beams. (**a**) The previous microstructure; (**b**) the improved cilium microstructure.

Stress simulation on the cantilever beams is carried out by ANSYS software. When the pressure along the *x*-axis acting on cilium is 1 Pa, the stress distribution on the cantilever beams is shown in Figure 8. The maximum stress on the cantilever of CVH can reach 28,344 Pa, and the improved microstructure can reach 80,864 Pa. The maximum stress of the improved microstructure is 2.85 times that of the previous one. In other words, the sensitivity is improved by 9.09 dB by the formula 20lg2.85.

**Figure 9:**
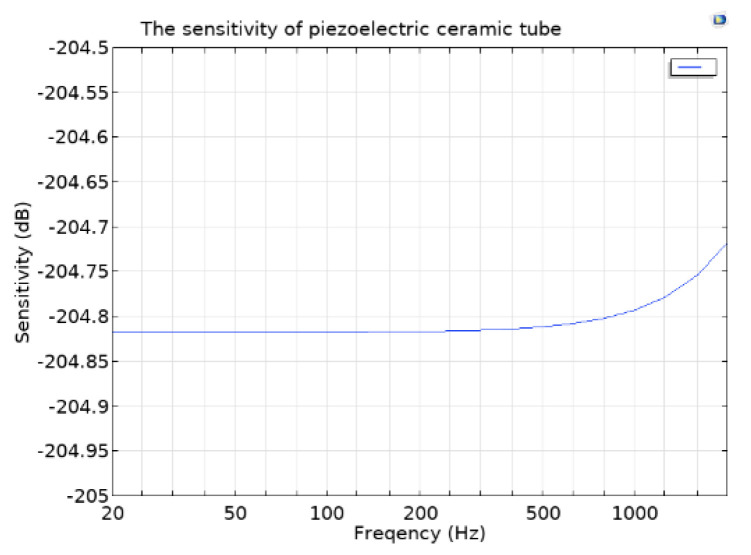
Sensitivity simulation of piezoelectric ceramic tube.

Simulation analysis of the piezoelectric tube sensitivity is carried out by COMSOL Multiphysics. The piezoelectric ceramic tube is put into the fluid domain with a 1 Pa incident pressure plane wave. The research frequency limits in the range of 20 Hz–2 kHz. The simulation result shows that the sensitivity of the piezoelectric ceramic tube can reach −204 dB, as shown in Figure 9.

### 5.2. Natural Frequency Analysis of Vector Channeland Piezoelectric Tube

**Figure 10:**
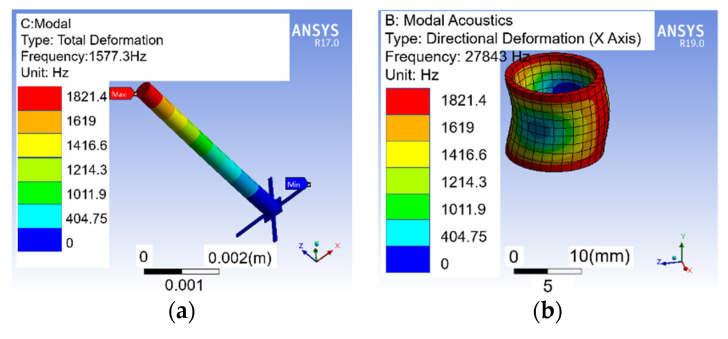
First-order modal analysis. (**a**) Vector channel; (**b**) scalar channel.

In order to ensure the working frequency range (20 Hz–1 kHz) of the composite hydrophone, the mode analyses are carried out. The simulation results are shown in Figure 10. The first-order mode of the vector channel can reach up to 1577.3 Hz, which maintains the natural frequency of the previous structure and meets the frequency range of ship noise (20 Hz–1 kHz) [24]. The radial vibration mode of the piezoelectric ceramic tube can reach up to 27,843 Hz.

## 6. Fabrication, Tests and Results

### 6.1. Fabrication

**Figure 11:**
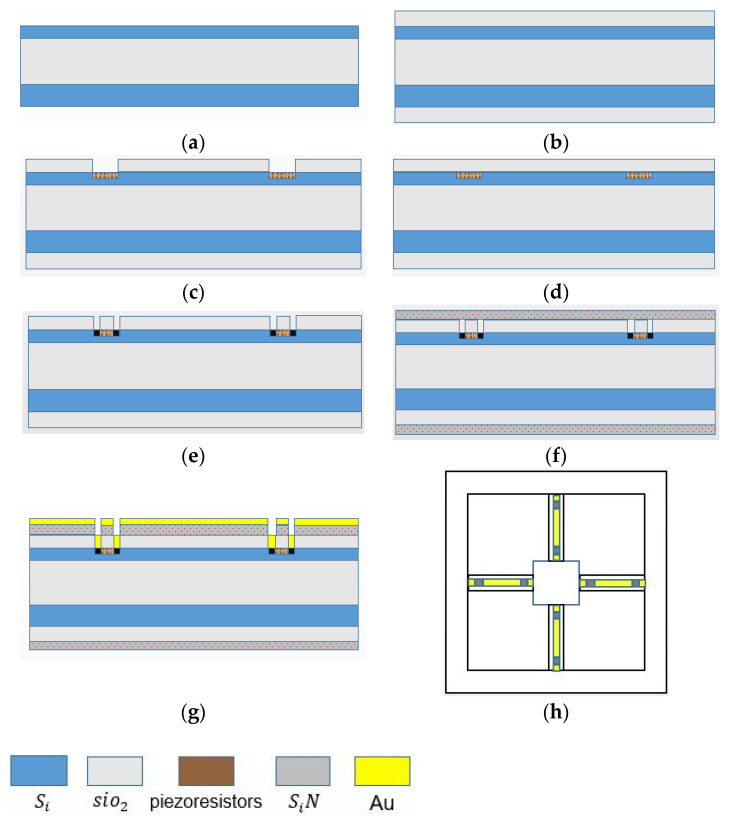
The processing technology of vector channel microstructure. (**a**) Preparation of SOI wafer; (**b**) oxidization to form silicon dioxide layer; (**c**) ICP etching and boron implantation; (**d**) re-oxidization; (**e**) ICP etching and heavily boron implantation; (**f**) silicon nitride depositing; (**g**) silicon nitride etching and Au deposition; (**h**) structure release.

**Figure 12:**
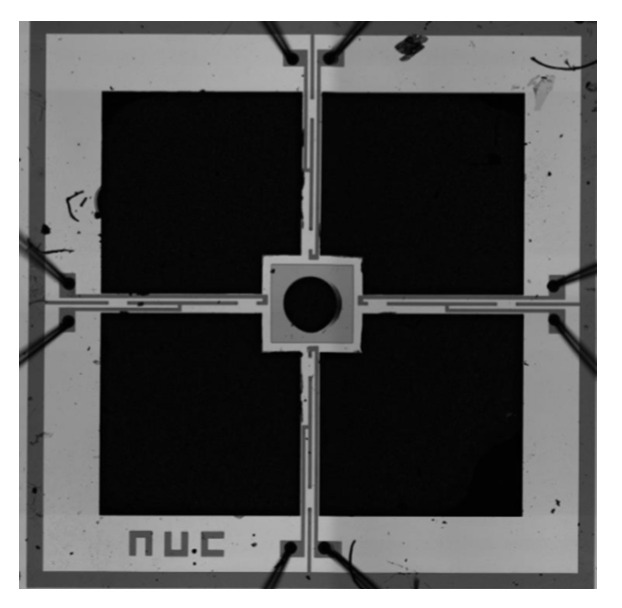
Optical microscope diagram of center block and cantilever beams.

The sensor micro-structure of the vector channel consists of the following three parts: four cantilever beams, central block, and cilium. Cilium is made of PE, and the cantilever beams and center block are fabricated by silicon MEMS technology. The main processes are shown in Figure 11, which include the following: (a) preparing 4-inch N-type SOI wafers with the electrical resistivity of 3~4 Ω⋅cm, the buried oxygen layer thickness of 2 μm, the substrate layer thickness of 400 μm, the device layer thickness of 40 μm; (b) oxidization at 950 °C, to form a 2000 Å silicon dioxide layer; (c) silicon dioxide etching via ICP and boron implantation (100 KeV, 4 × 10^18^ cm^−3^), to form the piezoresistors; (d) re-oxidization; (e) silicon dioxide etching and heavily boron implantation (100 KeV, 4 × 10^21^ cm^−3^), to form the P+ area; (f) silicon nitride depositing (1500 Å) via PECVD; (g) silicon nitride etching, Au deposition (1000 Å), and Au etching via iodine solution, to form the Wheatstone bridge; and (h) structure release. Finally, the optical microscope diagram of the center block and cantilever beams are shown in Figure 12.

**Figure 13:**
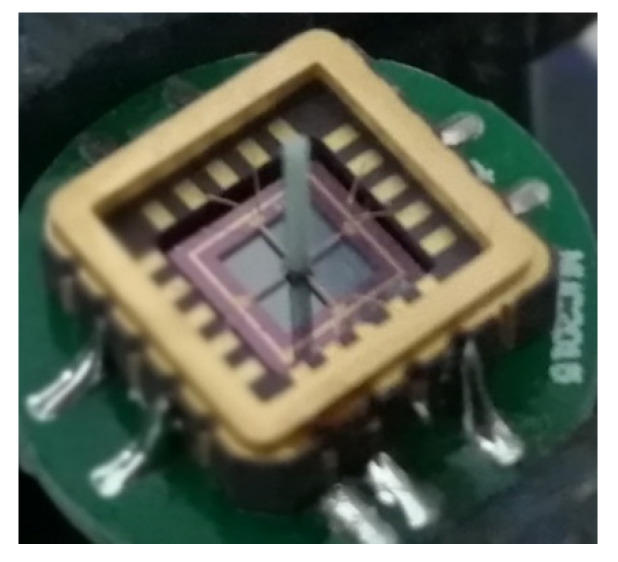
Secondary integration of cilium and central block.

**Figure 14:**
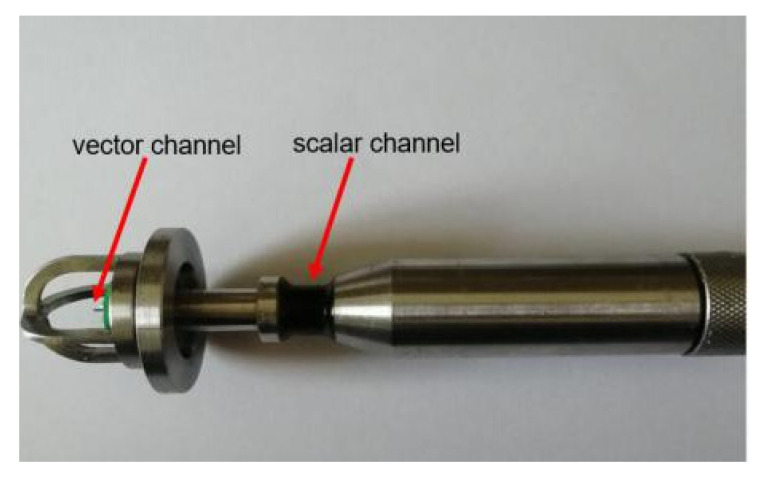
A physical picture of the composite hydrophone.

The secondary integration of cilium and the central block is performed by ultraviolet (UV) curing adhesive. Firstly, one section of the cilium is coated with UV curing adhesive, and then this section and the central block are fixed with an automatic integrated platform. Finally, those two parts are exposed under UV for three minutes. The microstructure after bonding is shown in Figure 13. The scalar channel and vector channel are installed on the metal shell, and the physical figure of the composite hydrophone is shown in Figure 14.

### 6.2. Tests and Results

**Figure 15:**
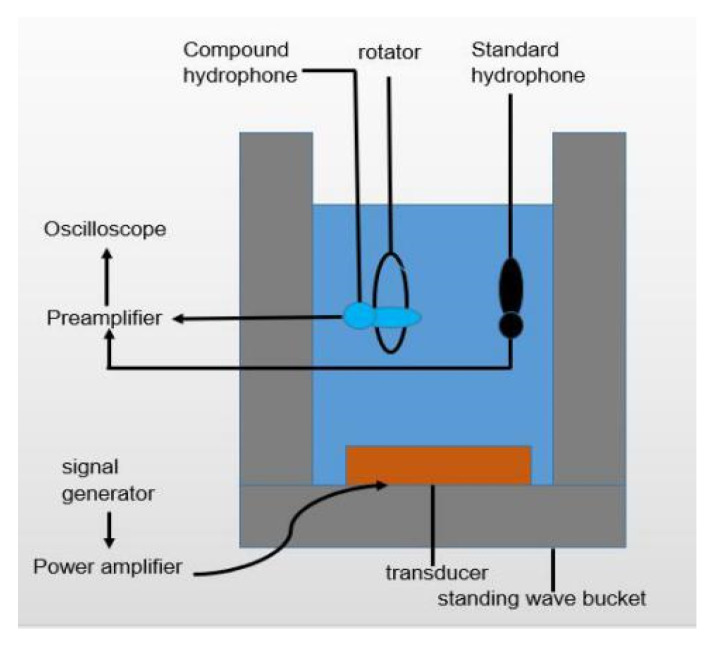
The schematic diagram of calibration system.

**Figure 16:**
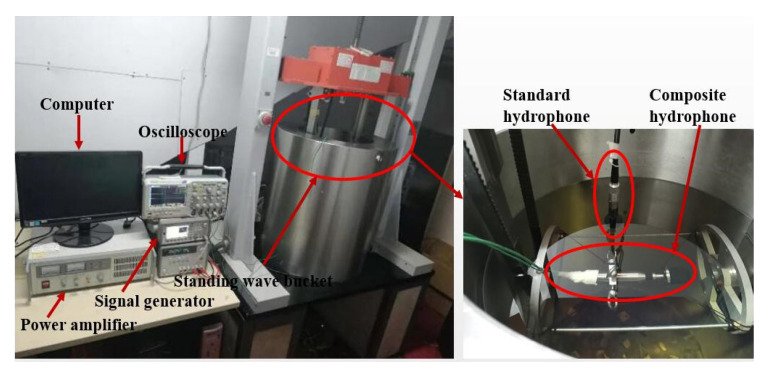
The composite hydrophone test system.

The tests on sensitivity and directivity are carried out by a calibration system, with a standard hydrophone. The system consists of a signal generator, power amplifier, transducer, preamplifier, oscilloscope, standing wave tube, and a vertical rotator. The standing wave tube provides a plane wave field, and a vertical rotator is used for testing the directivity of the hydrophone. The schematic diagram of the calibration system is shown in Figure 15. The excitation signal from the signal generator is amplified by the power amplifier, then converted into an acoustic signal by the transducer of the standing wave tube. The voltage output of the composite hydrophone and the standard hydrophone are recorded by the oscilloscope. The sensitivity is obtained by a free field comparison method. During the test, the standard hydrophone and the composite hydrophone are placed at the same height, below the liquid level, and the test frequency band is in the frequency range of 20 Hz–2 kHz, according to the frequency of 1/3 octave. The experimental test system is shown in Figure 16. The sensitivity of the composite hydrophone can be expressed as Formulas (9) and (10) [25], as follows:

(9) $$M_{v}=20\mathrm{lg}\left(\frac{e_{v}}{e_{o}}\frac{\sin kd}{\cos kd_{0}}\right)+M_{0}\;$$

(10) $$M_{s}=20\mathrm{lg}\frac{e_{s}}{e_{o}}+M_{0}$$

wherein, $M_{v}$ and $M_{s}$ are the sensitivity of vector channel and scalar channel, respectively; $M_{0}$ is the standard hydrophone sensitivity of −170 dB; $e_{v}$ and $e_{s}$ are the output voltage of vector channel and scalar channel, respectively; $e_{o}$ is the output voltage of standard hydrophone; d and $d_{0}$ are the distance from the water surface to tested hydrophone and standard hydrophone, respectively; *K* is the wave number.

**Figure 17:**
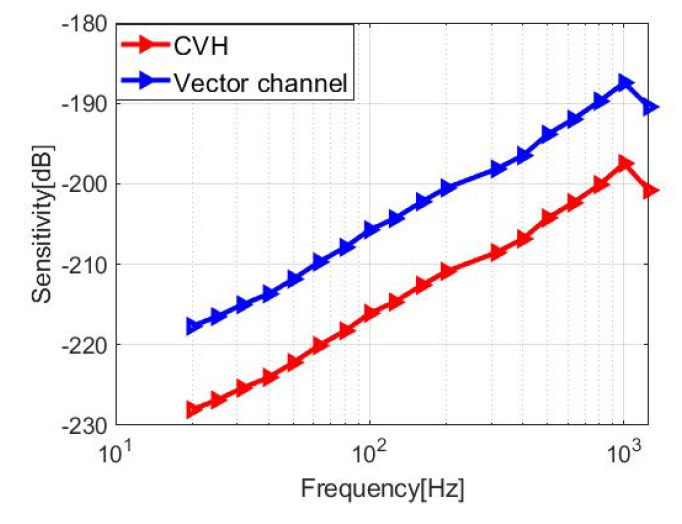
Sensitivity curves of CVH and vector channel (the improved cilium microstructure).

**Figure 18:**
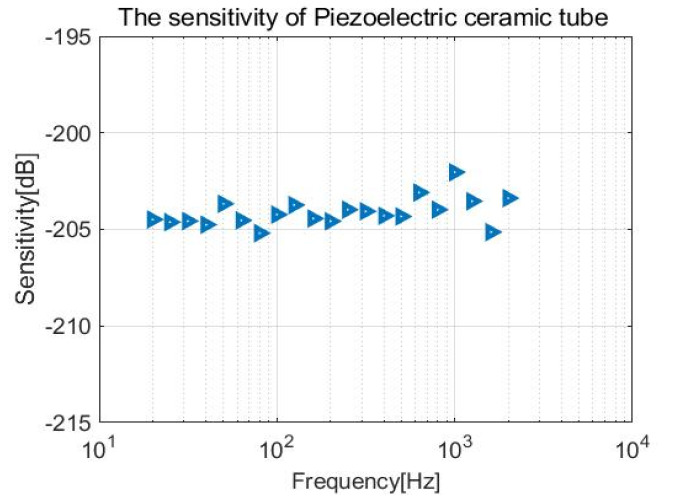
Sensitivity of scalar channel (the piezoelectric ceramic tube).

The sensitivity curves of the CVH and vector channel are shown in Figure 17. It can be observed that the sensitivity of the vector channel can reach 188 dB within the frequency band of 20 Hz–1 kHz, which follows with the theory and stimulation. As for the scalar channel, the sensitivity can reach 204 dB, with the test frequency range in 20 Hz–2 kHz. The sensitivity curve of the piezoelectric ceramic tube is shown in Figure 18.

**Figure 19:**
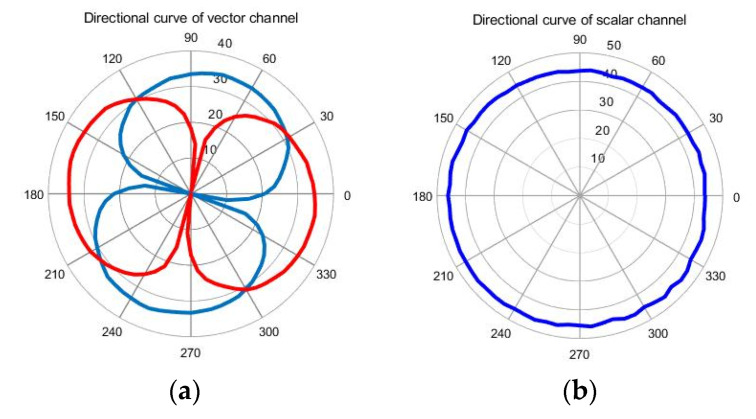
Directivity of composite hydrophone. (**a**) Vector channel at 500 Hz, (**b**) scalar channel.

The directivity determines the positioning performance of the composite hydrophone, which can be expressed as Formula (11) [25], as follows:

(11) $$L=20\mathrm{lg}\left(\frac{U_{\theta }}{U_{max}}\right)\;$$

wherein θ is rotation angle; $U_{\theta }$ is output voltage (vector channel and scale channel), and $U_{max}$ is the maximum output voltage. The directivity curves of the composite hydrophone are shown in Figure 19. Vector channel has a smooth and symmetrical eight-shaped directivity with the concave point depth above 30 dB. The scalar channel is directionless in the horizontal direction.

In order to confirm whether the problem of left–right ambiguity has been solved, the conjugate spectrum method [10] is used to estimate the azimuth of the target. The test is also completed in the standing wave tube. A continuous sinusoidal signal, with a frequency of 630 Hz, is transmitted, and the position of the composite hydrophone is adjusted so that the incidence angle of the sound wave is 180° and 270°, respectively. The data are collected by the NI acquisition card, and the sampling rate is set to 10 kHz.

**Figure 20:**
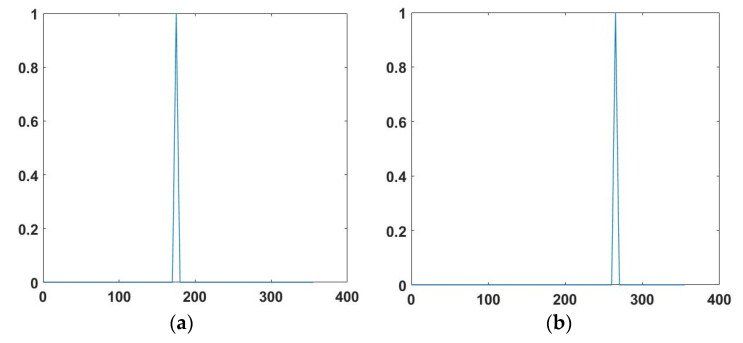
The azimuth estimation results, (**a**) DOA = 180°; (**b**) DOA = 270°.

The conjugate spectrum method is used to process the collected data. Figure 20 shows the test results of the acoustic incidence angle at 180° and 270°. Table 2 shows the directional results and error values of each angle. The error means the angle difference between the test results of the sound source location and the actual sound source location. We think that the positioning error mainly comes from the mechanical error when adjusting the position of the hydrophone. The test results show the error in 5°, and it can be concluded that the composite hydrophone has a precise orientation ability.

## 7. Discussion and Conclusions

In this paper, a new type of composite hydrophone is proposed and realized. The composite hydrophone consists of two channels. The vector channel is an optimized bionic cilium MEMS vector hydrophone, which improved the sensitivity by 9 dB, while maintaining a constant bandwidth compared to the previous work (CVH). The scalar channel is realized by a piezoelectric tube. The composite hydrophone can both acquire the scalar information and vector information of the underwater sound field. By combining two pieces of information in different ways, the composite hydrophone can solve the problem of left and right ambiguity when a single hydrophone is positioned underwater.

Due to the influence of the concave point depth, the accuracy of the azimuth measurement is the worst around *n* × 90° (*n* = 0–3). So, we quantitatively test the ability of the composite MEMS hydrophone in target orientation detection, when the sound wave enters into the device at 180° and 270°, respectively. The test results show that the positioning error is within 5°, which demonstrates an accurate positioning ability. Compared with the traditional scalar hydrophone array, the composite hydrophone is of great significance in the underwater positioning of a single hydrophone.

## Figures and Tables

**Table 1:** Sizes comparison between the two microstructures.

Sizes	CVH	This Work
Length of beam (*L*)	1000 μm	1000 μm
Width of beam (*b*)	120 μm	120 μm
Thickness of beam (*t*)	40 μm	40 μm
Half-length of center block’s side (*a*)	300 μm	300 μm
Diameter of cilium (*D*)	200 μm	500 μm
Height of cilium(*H*)	5000 μm	5500 μm

**Table 2:** Directional results and error values of each angle.

Frequency (Hz)	Incident Angle (°)	Location Results (°)	Error (°)
630Hz	180	176	4
	270	267	3
